# Short-term clinical effect of total hip replacement in the treatment of sequelae of septic arthritis of the hip: A retrospective study

**DOI:** 10.1097/MD.0000000000043269

**Published:** 2025-07-11

**Authors:** Cangxu Zhang, Qin Liu, Qian Kong, Dahai Zhang, Shicheng Xie

**Affiliations:** a Jining Medical College School of Clinical Medicine, Jining, China; b Affiliated Hospital of Jining Medical College, Jining, China; c Tancheng County Traditional Chinese Medicine Hospital, Jining, Shandong, China.

**Keywords:** clinical outcomes, sequelae, suppurative hip arthritis, total hip arthroplasty

## Abstract

Total hip arthroplasty in the treatment of sequelae of suppurative hip arthritis is very complicated. To observe the short-term efficacy and complications of total hip arthroplasty in the treatment of sequelae of suppurative hip arthritis and to improve the success rate and curative effect of operation, the clinical data of 32 patients with sequelae of suppurative hip arthritis who underwent total hip arthroplasty in our department from June 2013 to December 2020 were retrospectively analyzed. The age of hip suppurative infection was 2 to 17 years, with an average age of (9.8 ± 6.2) years. The average age was (35.2 ± 12.5) years. All the patients were treated by the direct lateral approach (Hardinge approach), and the acetabular and femoral sides were fixed with the biological prosthesis. All the 32 patients were followed up, and 2 patients had sciatic nerve palsy after operation, and the neurological symptoms recovered after conservative treatment for 1 month. One case of subtrochanteric osteotomy nonunion and femoral prosthesis loosening was treated with femoral lengthening stem revision. The clinical symptoms and hip joint activity of all patients were significantly improved after operation. The average Harris score was 43.3 ± 7.2 before operation and 88.6 ± 9.1 at the last follow-up after operation, and the difference was statistically significant (*P* < .05). The preoperative visual analogue scale was (7.1 ± 1.6) points, and it decreased to (1.1 ± 0.6) points at the last follow-up, and the difference was statistically significant (*P* < .05). Total hip arthroplasty in the treatment of sequelae of suppurative hip arthritis has a satisfactory short-term effect, which can effectively relieve pain and improve hip joint function after operation.

## 1. Introduction

Septic arthritis of the hip can occur in people of all ages, mostly children and adolescents. In developing countries, 5 to 20 people per 100,000 suffer from the disease, and the incidence has been increasing in recent years.^[1,2]^ Reasons include blood-borne infection, proximal femoral or iliac bone osteomyelitis invading the hip joint, infection in open joint injury, joint surgery or intra-articular injection of drugs, etc.^[3]^ The incidence and treatment effect are related to the economic and medical level, and the misdiagnosis rate is high. Early treatment maintains joint shape, restores function, and preserves cartilage. Usually, the prognosis is good. Unless the diagnosis and treatment are delayed, it may lead to permanent joint damage and long-term disability, followed by serious sequelae. It is one of the reasons for adult hip replacement. Total hip arthroplasty can provide patients with good joint function and a significant improvement in quality of life.^[4,5]^ The purpose of this study is to observe the short-term efficacy and complications of total hip replacement in the treatment of sequelae of septic arthritis of the hip, in order to improve the success rate and efficacy of the operation.

## 2. Methods

### 2.1. General information

From June 2013 to December 2020, the clinical data of 32 patients with sequelae of suppurative hip arthritis who underwent total hip arthroplasty were collected in the hospital. The diagnostic criteria are: (1) limited mobility of hip pain; (2) previous history of purulent infection of the hip joint, such as pain, high fever, sinus tract formation or surgical incision and drainage of sinus tract; abnormalities, such as pathological dislocation, missing femoral head, slipped epiphysis, stenosis and closure of femoral medullary canal, etc. This group of cases includes 19 males and 13 females, aged 46 to 69 years, with an average of (55.6 ± 8.7) years old, and the age at the time of hip suppurative infection was 2 to 17 years old, with an average of (9.8 ± 6.2) years old. The interval from initial infection to total hip replacement ranged from 19 to 52 years, with an average of (35.2 ± 12.5) years.

### 2.2. Preoperative preparation

All patients had a clear history of suppurative hip arthritis before operation, and the muscle strength of the gluteus medius was basically normal. Blood routine, erythrocyte sedimentation rate, white blood cell count, C-reactive protein, etc were within the normal range, excluding active infection. X-ray films of the hip joint and CT examination of the hip joint were performed to further clarify the condition of the affected hip joint, evaluate the size and deformity of the acetabulum and femoral medullary cavity, and preselect the appropriate prosthesis.^[6]^

### 2.3. Surgical method

All operations were performed by the same chief physician. All patients were under general anesthesia in a standard lateral position. The direct lateral approach (Hardinge approach) was chosen as the surgical approach, and the skin, subcutaneous tissue, and fascia lata were incised layer by layer. Care should be taken to protect the sciatic nerve, expose the acetabulum and proximal femur, and remove scar tissue and hypertrophic osteophytes around the hip joint. If it is difficult to expose, it is estimated that a subtrochanteric osteotomy is required, and the osteotomy is performed below the lesser trochanter to facilitate exposure. In this group of patients, 24 cases underwent subtrochanteric osteotomy, and the length of osteotomy was 2 to 6 cm, with an average of (3.2 ± 1.9)cm. During the operation, the joint fluid was collected for bacterial culture and drug sensitivity test, and the scar tissue around the hip joint was collected for pathological examination. The appropriate prosthesis was selected according to the intraoperative situation, and biological fixed prostheses were used on the acetabulum and femoral side. Check the tightness of the joint, wash with normal saline, stop bleeding, place a drainage tube, and close the incision layer by layer.

### 2.4. Postoperative treatment

According to the drainage volume, the drainage tube was removed 48 to 72 hours after operation. Immediately start the isometric contraction exercise of the quadriceps femoris of the affected limb on the day after the operation, and start the active hip joint extension and flexion exercise 1 day after the operation, use low-molecular-weight heparin sodium to prevent deep vein thrombosis 1 day after the operation, and prevent infection after 5 to 7 days with antibiotics sky. The time for getting out of bed and weight-bearing activities was determined according to the stability of the prosthesis during the operation. Postoperative 1 month, 3 months, 6 months, 12 months and subsequent follow-up every year, the hip joints were taken in anteroposterior and lateral radiographs.

### 2.5. Evaluation index

The occurrence of postoperative complications, preoperative and postoperative pain visual analogue scale scores, and Harris hip joint function scores were recorded. A score above 90 is excellent, 80 to 89 is good, 70 to 79 is fair, and below 70 is poor.^[7]^

### 2.6. Statistical methods

SPSS 24.0 software was used for statistical analysis of the data, and the measurement data were expressed as mean ± standard deviation (±s). The paired *t* test was used to compare the Harris hip scores before and after surgery, and the test level was two-sided α = 0.05.

## 3. Results

All 32 patients were followed up for 1 to 8 years, with an average of (4.6 ± 3.2) years. Sciatic nerve paralysis occurred in 2 cases after operation, and the neurological symptoms recovered after 1 month of conservative treatment. In 1 case, the subtrochanteric osteotomy was nonunion, the femoral prosthesis was loose, and the femoral prosthesis was extended and revised. There was no prosthetic dislocation, loosening, sinking, deep vein thrombosis and other complications, and no recurrence of infection was found during the follow-up period. The postoperative clinical symptoms and hip joint mobility of all patients were significantly improved. The average Harris score (Table 1) was (43.3 ± 7.2) before operation and (88.6 ± 9.1) at the last follow-up after operation, and the difference was statistically significant (*P* < .05). The preoperative visual analogue scale score was (7.1 ± 1.6) and decreased to (1.1 ± 0.6) at the last follow-up after operation, and the difference was statistically significant (*P* < .05).

**Table 1:** Age and preoperative and postoperative scores of patients in the 2 groups.

Score	Male/female	Age (yr)	Preoperative score (points)	Postoperative score (points)
Harris score	19/13	55.6 ± 8.7	43.3 ± 7.2	88.6 ± 9.1
VAS score	19/13	55.6 ± 8.7	7.1 ± 1.6	1.1 ± 0.6

VAS = visual analogue scale.

**Figure 1.**
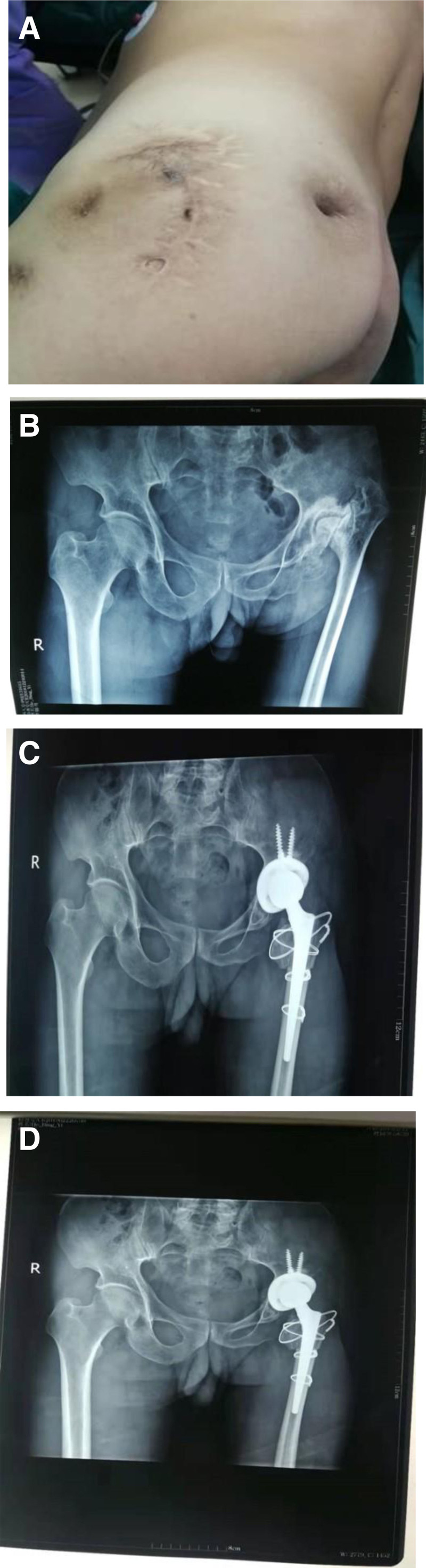
(A) Preoperative photos showed multiple wounds and sinus scars. (B) Preoperative radiographs showing missing left femoral head. (C) Postoperative radiographs, subtrochanteric osteotomy. (D) Radiographs 1 year after surgery showed that the osteotomy had healed.

Typical case: a 68-year-old male with pain in the left hip joint and limited mobility for >55 years, which aggravated for 2 years. He had a history of septic arthritis of the left hip. Irregular wound scars were seen on the left hip, without redness and swelling, the hip joint was limited in motion, and the affected limb was shortened by about 6 cm. During the operation, a biological acetabular cup and Wagner cone femoral stem were used, and a 2.5 cm subtrochanteric osteotomy was performed (Fig. 1).

## 4. Discussion

### 4.1. Characteristics of sequelae of septic arthritis of the hip

Septic hip osteoarthritis, the most common type of septic arthritis in children, has a predominantly methicillin-sensitive *Staphylococcus aureus* pathogen, but the percentage of culture-negative infections ranges from 16.7% to 78.4%.^[8–12]^ Inadequate treatment, especially in younger children with immature skeletal development, leads to progressively worsening deformities, including osteoarthritis and hip dysplasia secondary to disruption of the cephaloacetabular relationship; LUO Y et al further pointed out that this type of deformity can be manifested as pathologic dislocation, absence of femoral head, joint stiffness, unequal length of both lower limbs and other deformity.^[13]^ It may be accompanied by localized changes such as anatomical variations of the femur (e.g., abnormal anterior inclination angle, dysplasia), periprosthetic soft tissue scar contracture, and superior displacement of the greater trochanter, which may ultimately lead to systemic deformities such as compensatory scoliosis of the lumbar spine and internal and external derangement of the knee joints; Aggarwal A and Aggarwal AN reported that sometimes there is a potential risk of infection, even if the inflammatory indicators such as C-reactive protein and erythrocyte sedimentation rate are normal, the infection cannot be completely ruled out.^[14]^ This suggests that clinical assessment needs to be combined with multidimensional indicators for comprehensive judgment.

### 4.2. Prosthesis selection

Choosing a suitable reconstructive prosthesis is the key to the success of the operation. The prosthesis needs to have the characteristics of good compatibility with human tissue, good fit, stability, and durability. For those without dislocation and normal femoral medullary cavity, common prostheses can solve the problem. Younger patients who have been used for a long time may have revisions later, and biological prostheses are preferred.

In general, the type of implant and the presence or absence of osteotomy (e.g., femoral osteotomy) in total hip arthroplasty (THA) have an impact on surgical outcomes, but the exact effect varies depending on the individual patient, surgical indications, and choice of technique. Different implant materials and fixation methods can influence, for example, ceramic interfaces have a low wear rate and are suitable for young, active patients; metal-polyethylene may lead to osteolysis due to wear particles. Cemented type of fixation is suitable for osteoporotic or elderly patients with good early stability but possible cement-related complications. Uncemented (biologic) fixation relies on bone growing into the biologic fixation and is more suitable for younger, well-boned patients with superior durability.

Osteotomy is usually used in complex cases (e.g., severe deformity), and it improves anatomical alignment, restores hip joint centering, and reduces prosthetic stress. However, there are some disadvantages such as prolonged operative time, increased bleeding, and poor healing of the osteotomy. In this study, because the patients were not elderly patients, biologic fixed prosthesis was used in both acetabular and femoral side. The patients in this study recovered well with few complications and there was no significant difference in postoperative recovery and improvement in Harris score between osteotomized and non-osteotomized patients.

Compared with congenital hip dysplasia, septic hip arthritis is significantly more difficult to release due to its specific pathologic changes, and even in the case of relatively mild dislocations, subacromial osteotomies are often required, which sometimes become the only feasible option to achieve joint reset.^[15]^

To complicate matters further, since septic hip arthritis is often complicated by osteomyelitis of the proximal femur, which in turn leads to pathologic narrowing of the bone marrow cavity, this calls for surgical treatment with the selection of a special femoral stem prosthesis that is highly compatible with the anatomical structure of the deformity, such as Wagner cone, S-Rom, and Chunli 130 with better fit.^[16–18]^ Camere et al reported that a Wagner Cone stem was used in the treatment of total hip arthroplasty for a minimum of 20-year follow-up study to evaluate the prosthetic survival rate, clinical function and imaging results after surgery.^[18]^ The 20-year survival rate of the prosthesis was 97% (95% Cl, 94.4–99.6), as well as excellent performance in terms of clinical functional assessment and imaging results, which provides an important evidence-based basis for long-term prognosis of such complex cases provides an important evidence-based rationale.

In patients with residual joint deformity from septic hip arthritis, the intraoperative selection of a small-sized prosthesis (with a minimum of a 38-sized cup) has become a routine technical requirement due to the specific pathologic features, including acetabular dysplasia, reduction of the acetabular volume, and weakness of the acetabular wall bone.^[19]^ Among the 32 cases in this group, 24 cases underwent subtrochanteric femoral osteotomy due to severe deformity, of which 16 cases were reconstructed with Wagner cone femoral prosthesis and 8 cases were reconstructed with Chunli 130 femoral stem. However, it should be pointed out in particular that one patient had a postoperative subtrochanteric osteotomy without healing with loosening of the femoral prosthesis, which was eventually remedied by the Chunli 160 femoral lengthening stem revision surgery.

### 4.3. Preoperative determination of difficulty of exposure and subtrochanteric osteotomy

When evaluating the difficulty of surgical visualization of posterior deformities in septic hip arthritis, multiple factors need to be taken into account: first, the initial judgment is based on the extent of scarring and the size of the sinus tracts; the more extensive the scarring and the more numerous the sinus tracts, the more difficult the surgical visualization will be. Cai D and other studies^[1]^ further pointed out that the mobility of the hip joint is another key assessment index: joint stiffness or ankylosis not only reflects the abnormality of the bony structure, but also suggests the existence of severe soft tissue contracture and loss of compliance, while good mobility indicates that the soft tissue conditions are relatively ideal; in addition, it should be combined with the direction of activity limitation, the degree of dislocation and the horizontal offset of the femoral neck (Offset). In addition, the direction of limitation of motion, the degree of subluxation, and the horizontal offset (Offset) of the femoral neck should also be taken into account: a larger Offset value usually means better soft tissue conditions, which is favorable for surgical operation, whereas a smaller Offset value suggests a severe contracture of the soft tissues, which will lead to difficulties in femoral distraction and increase the difficulty of surgical exposure significantly.

Subtrochanteric osteotomy is an important adjunctive technique in total hip arthroplasty for the treatment of the sequelae of septic hip osteoarthritis, primarily for the resolution of complex anatomical deformities and mechanical abnormalities resulting from the sequelae of infection. Septic hip arthritis results in destruction and resorption of the femoral head, with residual shortening of the femur or acetabular deformity, which may be accompanied by joint ankylosis or fibrotic healing. Subtrochanteric osteotomy restores biomechanical alignment of the hip joint and corrects the deformity by osteotomizing the bone in the subtrochanteric region (distal to the lesser trochanter) and restructuring the proximal femur. In septic arthritis, the proximal femur is often inversion and anterior tilt angle is abnormal. Subtrochanteric osteotomy can adjust the line of force of the femoral stem and normalize the line of force of the lower limb after implantation of the prosthesis. After osteotomy, the distal femur can be rotated or translated to better align the femoral prosthesis with the acetabular prosthesis and reduce uneven joint stress. Lower extremity length discrepancies can also be addressed by first loosening contractured soft tissues through osteotomy and then gradually adjusting lower extremity length. Subtrochanteric osteotomies can also relieve contractures of the gluteus medius and external rotator muscle groups, reducing the risk of postoperative dislocation. In addition, it reduces the difficulty of prosthesis implantation and avoids femoral splitting or perforation caused by forced marrow expansion.

### 4.4. Acetabular exposure and proximal femur exposure

In the process of acetabular side surgical exposure, Zhao E and other scholars^[20]^ suggested that the tight adductor muscle can be cut off preoperatively, and the broad fascia with severe contracture needs to be partially cut off and loosened;^[21]^ at the same time, a sufficiently long surgical incision should be made to remove the intra-articular scar tissue thoroughly, and promote the femur posteriorly by loosening the joint capsule and the rectus femoris muscle retractor head at the anterior side, and the adequate loosening of the iliopsoas muscle is also indispensable; Hozack WJ et al^[22]^ also pointed out that selective removal of a part of the bony capsule will significantly improve the surgical visualization when there are more bony bones present.^[20]^

In the process of acetabular side exposure, special attention should be paid to the following: a reasonable sequence of release should be selected according to the different pathological states of the hip joint, avoiding excessive release leading to joint instability or limb lengthening while releasing, evaluating and treating synchronously, especially preventing intraoperative fracture triggered by violently tractioning the femur under the state of soft-tissue tension^[21]^; in cases of joint ankylosis and fixation deformity, the anatomical structure should be identified precisely, and be vigilant against accidental injury to the ilium or pubic bone, so as to improve the field and assist anatomical positioning. In cases of ankylosis and fixation deformity, it is necessary to identify the anatomical structures accurately, be alert to accidental injuries to the ilium or pubis, and perform a greater trochanteric osteotomy to improve the operative field and assist in anatomical localization if necessary.^[22]^

In the process of proximal femur exposure, scar tissue, iliopsoas muscle and gluteus maximus reflex head and other structures need to be gradually loosened along the proximal femur in order to pry out the proximal femur smoothly; for high dislocation cases, due to the significant increase in the difficulty of loosening, exposure and repositioning, subminiar osteotomy is often required; when the preoperative evaluation suggests that subminiar osteotomy is required, osteotomy can be completed in priority, and then the acetabulum and the proximal femur can be exposed subsequently, which can effectively reduce the difficulty and improve the operational safety. This can effectively reduce the difficulty and improve the safety of the operation.

### 4.5. On bilateral lower extremity inequality and postoperative rehabilitation exercises

In total hip arthroplasty, the difference in the length of both lower limbs is a key factor affecting the recovery of hip function after surgery,^[23]^ and factors such as lumbar spine flexibility, pelvic tilt, and knee joint compensatory ability need to be comprehensively assessed before surgery, especially for patients with poor lumbar spine flexibility, complete correction of the unequal length of the lower limbs may lead to postural compensatory imbalance, which may lead to complications such as lower back pain and knee extensor; attention needs to be paid to the one-time during the operation. During surgery, it is necessary to pay attention to the one-time limb lengthening should not be too much, in order to avoid the difficulty of reset and the risk of vascular and nerve injury, and at the same time, it should be recognized that the osteotomy under the small rotor may lead to the shortening of the limb, which may lead to the new problem of unequal lengths, therefore, the surgeon should carry out a comprehensive preoperative assessment, and accurately control the length of the osteotomy during the operation, so that the lower extremities can realize the relative length of the lower extremities as much as possible under the premise of ensuring the safety of the operation. In this case, through precise preoperative planning and intraoperative operation, the difference between the lengths of the 2 lower limbs was successfully controlled within the ideal range of 1 cm.

Sciatic nerve injury is a serious but relatively uncommon complication in total hip arthroplasty and hip osteotomy. Over lengthening of the lower extremity as a result of resetting the femoral head to the true socket can directly pull on the sciatic nerve (especially in patients with a previous hip contracture or shortening). The following factors may also contribute to sciatic nerve injury, improper surgical maneuvering, general proximity of the sciatic nerve in the posterior lateral approach, improper placement of the pulling hooks or during electrocoagulation for hemostasis, piercing during acetabular filing or femoral reaming, and direct compression or cutting of the nerve may directly injure the nerve. In this study, which was a direct lateral approach, the cause of sciatic nerve palsy was related to the pulling of the pulling hook when exposing the field of view. The faster recovery of the nerve in the subsequent patients indicates that the possibility of pulling injury is high, and the cause of instrument cutting injury during direct osteotomy is less. Secondly, abnormal prosthesis position such as posteriorly tilted or protruding acetabular cup may directly compress the sciatic nerve. An abnormally long femoral stem or anterior tilt angle leads to nerve pull or impingement. In addition, postoperative hematomas compress the sciatic nerve, and the sciatic nerve blood supply (e.g., branches of the inferior gluteal artery) is compromised intraoperatively.

The duration of weight bearing is determined by the stability of the prosthesis, waiting for the bone to grow in (usually 6 weeks). Partial weight bearing of 20% to 30% of body weight is recommended in the early postoperative period, with a gradual transition to full weight bearing after 6 weeks. If the intraoperative prosthesis is stable and the bone quality is good, and the patient’s body weight is small, early full weight bearing is possible, but individualized assessment is required. Patients have postoperative postural restrictions (especially in the first 3–6 weeks during the high-risk period), avoiding hip flexion >90°, pronation over the midline, internal rotation (e.g., stooping, bending over to pick up something), limiting hip extension and external rotation (e.g., keeping the knee in neutral when getting in and out of bed), and using pillows or abductor cushions to keep the hip joint abducted by 15° to 30° (especially important during sleep). Postoperative day 1 bed ankle pump training, quadriceps contraction, get out of bed usually within 1 to 3 days, 2 to 4 weeks walking with the help of a walker, 6 weeks later gradually walk on their own.

In summary, the short-term curative effect of total hip arthroplasty in the treatment of sequelae of septic arthritis of the hip is satisfactory, and postoperative pain can be effectively relieved and hip joint function can be improved. However, the operation is complicated, and the key points of the operation technique must be strictly grasped, and sufficient soft tissue release during the operation is the key to the success of the operation.

This study has some limitations. First, from the perspective of study design, this study adopted a retrospective study design with a relatively low grade of clinical evidence-based evidence, making the reliability and generalizability of the study findings somewhat limited. Therefore, future studies may consider adopting a prospective study design to improve the level of evidence and scientific validity of the study. Second, in terms of sample size and study scope, single-center regional clinical data, the inclusion of sample size is relatively small, and this small-sample, short-follow-up study design may not be able to comprehensively real situation and long-term prognosis. Therefore, multi-center and large sample size studies can be conducted afterwards, and more regional large tertiary or specialty hospitals can be selected to expand the sample size.

## Author contributions

**Conceptualization:** Shicheng Xie.

**Data curation:** Dahai Zhang.

**Project administration:** Qin Liu.

**Resources:** Qin Liu.

**Software:** Qin Liu.

**Supervision:** Qian Kong.

**Validation:** Qian Kong.

**Writing – original draft:** Cangxu Zhang.

**Writing – review & editing:** Shicheng Xie.
